# The roles of nurses in supporting health literacy: a scoping review

**DOI:** 10.3389/fpubh.2023.1022803

**Published:** 2023-08-16

**Authors:** Angga Wilandika, Moses Glorino Rumambo Pandin, Ah Yusuf

**Affiliations:** ^1^Faculty of Nursing, Universitas Airlangga, Surabaya, Indonesia; ^2^Faculty of Health Sciences, Universitas Aisyiyah Bandung, Bandung, Indonesia; ^3^Faculty of Humanities, Universitas Airlangga, Surabaya, Indonesia

**Keywords:** health literacy, health information, patient health condition, nursing, nurse

## Abstract

**Introduction:**

The importance of health literacy in achieving optimum health is highly significant, particularly in the nursing profession where it is an integral part of the roles and functions of nurses. Therefore, this scoping review aims to describe the roles of nurses in promoting patient health literacy and identify the determinant factors of health literacy in nursing practices.

**Methods:**

An integrative search was conducted through four databases, namely, ScienceDirect, ProQuest, SAGE Journal, and PubMed, using various keyword combinations such as “health literacy,” “health information,” “patient health literacy,” “patient literacy,” and “nurses.” Furthermore, the inclusion criteria employed were peer-reviewed articles focused on the nursing profession, explicitly discussing health literacy related to nursing, and including original studies, such as cross-sectional, quasi-experimental, and qualitative studies. The selected review articles were all published between 2017 and 2022.

**Results:**

In total, 13 articles met the criteria and were applied in this scoping review. Most of these discuss health literacy related to nursing practice in clinical and community settings, as well as educational institutions. Health literacy is an essential aspect of professional nursing practice. Consequently, the supportive roles of nurses include acting as caregivers, facilitators, and educators to help patients overcome their literacy limitations and attain improved wellbeing.

**Conclusion:**

Nurses can improve the health literacy skills of patients by making health information related to their illnesses easier to access, understand, evaluate, and use. They must also recognize various factors influencing health literacy and use the factors as opportunities to optimize health literacy improvement. A health literacy approach can be applied by nurses to solve health problems and improve the quality of care for patients.

## Introduction

Health literacy is a concept that has been in existence for a long time but is relatively new in professional nursing practice. It is interpreted as health promotion (1), which is one of the goals of nursing science. This activity aims to maintain or improve individuals, groups, and communities (2). According to the American Nurses Association (ANA) (3), nurses must participate in ensuring and promoting the health of individuals, groups, communities, and society in general. Therefore, nurses should be well-equipped to protect, promote, and optimize the health and ability of everyone.

Nurses must understand health literacy as a concept that contributes to the health attitudes and behavior of individuals (4) in order to reduce health information gaps for patients (5). Limited health literacy can lead to health disparities and poor quality of care (6). Therefore, nurses and other healthcare providers need to be mindful of supporting health literacy to reduce existing disparities (7, 8). Patients with adequate health literacy are competent in paying attention to their health, families, and communities (9, 10). Good health literacy will improve healthy lifestyle behaviors and increase individual capacity to manage disease conditions (11–13). Meanwhile, limited health literacy will affect behaviors related to self-management of diseases, as well as health outcomes and costs (14, 15).

Based on the perspective of healthcare workers, health literacy can be described as a skill involving knowledge, motivation, and the ability to process information (16). This term is seen as a means of enabling individuals to access, understand, evaluate, and use health information to improve their wellbeing (17). Similarly, Guzys et al. (18) defined health literacy from the perspective of healthcare providers by emphasizing the knowledge and skills needed to prevent diseases and improve health in everyday life.

Health literacy skills in professional nursing facilitate effective communication between professional care providers and the public or clients. Carrying out this effective communication based on appropriate health information will enable patients to make the best decisions concerning their health problems (19, 20). Patients who make decisions based on health literacy are highly valued as they tend to be well-informed. Due to this, health literacy is defined as the process of obtaining and using correct information in making decisions related to health. It can be seen as a complex concept and requires a greater understanding of the nursing profession (21). Nurses need to understand that no one is entirely health literate. Therefore, they should have the necessary skills to help patients utilize information and correct misconceptions about diseases resulting from misinformation obtained from other sources (22). The general understanding of health literacy in nursing is of utmost importance, and more exploration is necessary in this area (23).

The enhancement of health literacy through health optimization is an integral aspect of professional nursing practice. Nurses, as healthcare professionals, play a vital role in addressing the issue of health literacy by comprehending the various forms of health literacy and providing quality care for patients. However, nurses understanding of health literacy and their associated supportive roles are still limited (23). The nature of health literacy in nursing practice needs to be explained to provide nurses with a clear understanding of how to utilize this concept. Therefore, this review aims to describe the roles of nurses in improving patient health literacy and identify the determinant factors that influence health literacy.

## Methods

### Protocols

Scoping reviews have been used to identify knowledge gaps, determine the scope of a body of literature, or clarify concepts (24). In this study, a scoping review approach was employed to collate articles focused on health literacy in nursing. An integrative search was conducted, and the results were presented following the guidelines of the Preferred Reporting Item for Systematic Reviews and Meta-Analysis (PRISMA) Extension (25). Therefore, the review question was formulated as “What are the role of nurses and the factors influencing it to increase health literacy within the scope of nursing?” Sub-questions explored to address the primary study question included the following:

- “What is the role of nurses in strengthening health literacy?”
- “What are the determinant factors influencing health literacy in nursing practice?”

#### Information sources and search strategy

In consultation with the study team, an expert information specialist conducted comprehensive literature searches through ScienceDirect, ProQuest, SAGE Journal, and PubMed to identify potentially relevant documents. These four databases were searched to fulfill the aims outlined above, using search terms specific to the context of health literacy in nursing. The searches were performed using various keyword combinations and Boolean operators, incorporating synonyms of “health literacy” or “health information,” along with “patient health literacy,” “patient literacy,” and “nurses.” Furthermore, the article search menu in all databases was enabled based on the nursing subject. The entire search terms were based on Medical Subject Headings (MeSH). The articles included in this review were limited to those peer-reviewed in the English language.

### Eligibility criteria

Due to the vast literature obtained, the analysis conducted was restricted to the articles published in the last 6 years, from 2017 to 2022. The study team initially screened articles by reading the title and abstract and then reviewed the full text of relevant articles to determine their eligibility for inclusion. The inclusion criteria consisted of articles that (1) focused on individuals in the nursing field, with study subjects including nurses, nurse educators, and nursing students; (2) explicitly discussed health literacy related to nursing; and (3) included original studies, such as cross-sectional, quasi-experimental, qualitative, and mixed-method studies. Meanwhile, the exclusion criteria applied were as follows: (1) articles focused on health literacy involving various healthcare workers except for nurses; (2) articles that do not discuss health literacy about nursing; and (3) literature reviews, dissertations, editorials, commentaries, and other expert opinions.

#### Study selection process

Articles were selected based on inclusion criteria through predetermined data-based searches. Those found during the search were added to the bibliography manager software (Mendeley). Duplicates were excluded through the automatic duplication removal process in Mendeley's tool. Articles not identified by the software were further examined and manually removed.

During the review process, two reviewers independently filtered and assessed each article using two stages based on the eligibility criteria. The first stage involved reviewing the title and abstract, while the second stage included a thorough examination of the full-text articles selected from the first stage. Any article deemed relevant by at least one reviewer progressed further in the review process. Subsequently, two independent blind readers checked and scored the Materials and Methods as well as Results sections, and only articles they both considered relevant were included in this study. To ensure consistency and accuracy in the selection process, Cohen's Kappa and percent agreement were used to calculate inter-rater reliability for both titles/abstract and full-text articles (26, 27). The Altman benchmark scale was employed to interpret the agreement degree of the Kappa value: < 0.20 (poor), 0.21–0.40 (fair), 0.41–0.60 (moderate), 0.61–0.80 (good), and 0.81–1.00 (very good) (26). A Kappa value >0.41 was considered acceptable (27). All disagreements between the two reviewers were discussed and resolved until a consensus was reached, and a third opinion was sought from a senior reviewer when unanimity was required.

### Data extraction and analysis

After selecting the articles, their data were entered into a spreadsheet for extraction and charting. For those in form of scoping reviews, data on study characteristics, such as years of conduct, aims, design, and subjects, were abstracted and are presented in Table 1. The years of publication were limited to 2017–2022 according to the eligibility criteria. The design selected was primarily cross-sectional, and only one appeared quasi-experimental. To align with the study aims and formulated questions, the data were also extracted based on the aims and subjects related to the role of nurses and determinant factors of health literacy in nursing. In the next stage of analysis, all publications were screened for a second time to verify whether these issues were being addressed in the included articles.

**Table 1:** Characteristics of the reviewed studies.

**Author, year**	**Aims**	**Study Design**	**Subjects**
Coşkun and Bebiş (11)	To determine the impact of health promotion on developing healthy lifestyle behaviors and health literacy in nursing students.	Quasi-experimental study	133 nursing students
Kim and Oh (28)	To identify the relationship between e-health literacy and health promotion behavior through social media related to health information, health information-seeking behavior, and self-care agency.	Cross-sectional study	558 nursing students
Mosley and Taylor (12)	To identify health literacy-based patient learning curricula and strategies.	Cross-sectional study	15 nursing educators and 53 nursing students
Shiferaw et al. (29)	To examine internet use and electronic health literacy skills among university students	Cross-sectional study	236 nursing students
Wittenberg et al. (20)	To assess nurse communication and health literacy in patients	Cross-sectional study	74 nurses
Ayaz-Alkaya and Terzi (30)	To determine health literacy and the factors influencing it in nursing students.	Cross-sectional study	303 nursing students
Nesari et al. (19)	To assess the knowledge and experience of registered nurses with health literacy practices.	Cross-sectional study	190 nurses
Turan et al. (31)	To examine the effect of e-health literacy on students' healthy lifestyle behavior.	Cross-sectional study	232 nursing students
Koduah et al. (32)	To assess the knowledge of health literacy and the factors influencing nurses.	Cross-sectional study	876 nurses
Munangatire et al. (33)	To explore nursing students' understanding of health literacy concepts and health practice.	Cross-sectional study	205 nursing students
Yusefi et al. (34)	To assess health literacy status and its relationship with quality of life among hospital nurses.	Cross-sectional study	185 nurses
Kim and Oh (35)	To identify nurses' perspectives on health literacy and education in elderly patients.	Qualitative study	16 nurses
Yang (36)	To identify the relationship between health literacy competencies and patient-centered care by clinical nurses.	Cross-sectional study	180 nurses

The team read and assessed the entire results, which were then summarized descriptively and compared in the data extraction sheet to identify common themes. Two reviewers independently identified themes for synthesis using thematic analysis that matched the study aims, and all discrepancies were resolved through discussion. Data descriptions indicating statements related to the role of nurses in literacy were grouped, and all results that showed factors related to health literacy in nursing were reviewed. These were organized into two main categories based on the sub-review questions: (1) the role of nurses in promoting health literacy and (2) factors influencing health literacy in nursing.

## Results

### Search result

**Figure 1:**
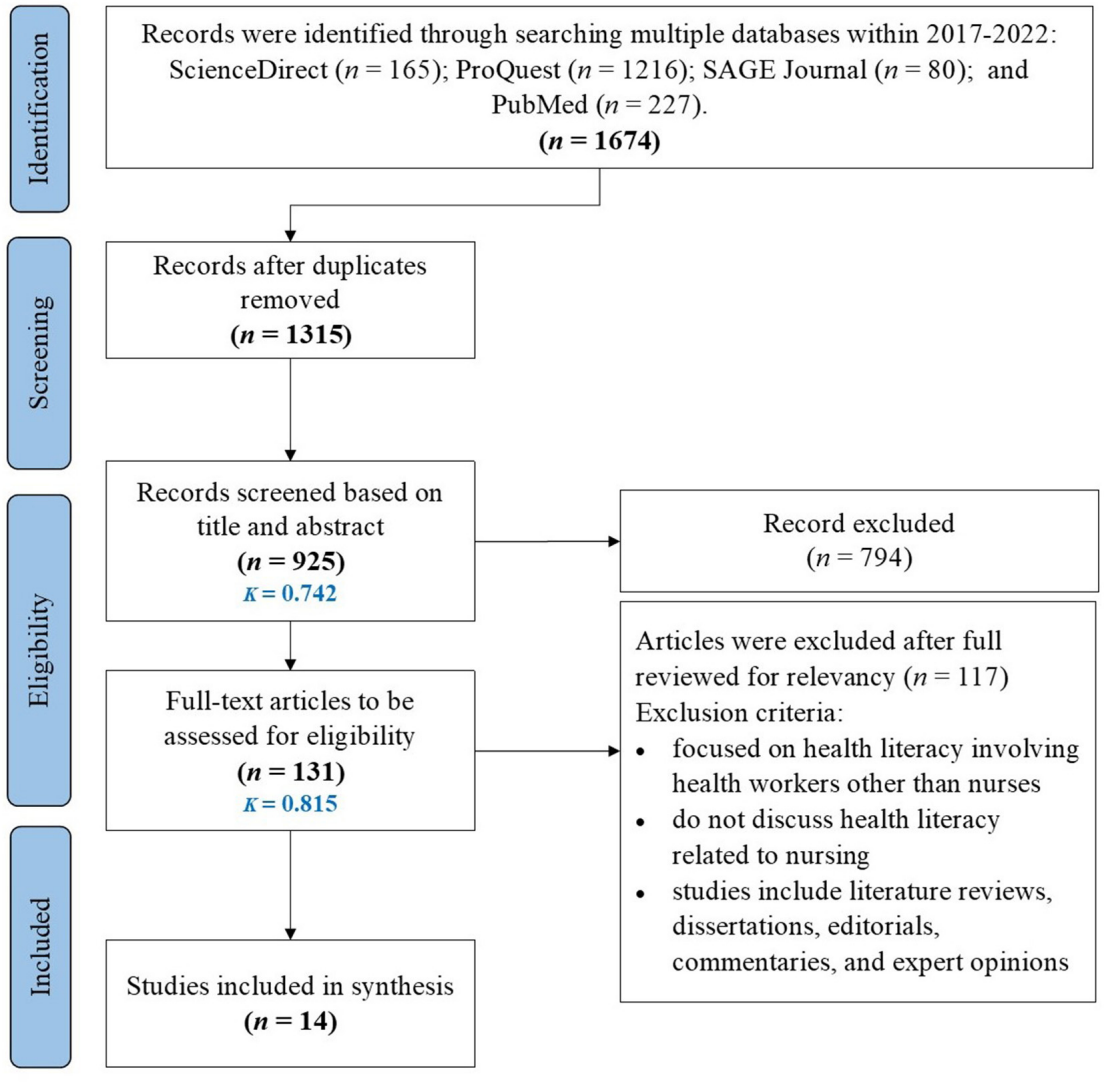
PRISMA scoping review flow diagram. Selection of sources of evidence.

The PRISMA-ScR flow diagram (25) presented in Figure 1 depicts each step of the article selection process, including the specific rationale for the exclusion of full-text articles. An initial search through four electronic databases and review article references yielded 1,674 results, which were reduced to 1,315 after removing duplicates. Two independent reviewers evaluated 925 articles in a two-step screening process. First, title and abstract screening for key terms, including health literacy, health literacy in nursing, nurses' roles, and factors related to patient health literacy, led to the exclusion of 794 records. Second, the remaining 131 articles were further assessed to determine their eligibility. From this, 117 articles were eliminated for the following reasons: 55 were not original studies, 39 did not align with the nursing scope, and 23 did not explicitly address health literacy in nursing; then, the 14 articles left were included in the final examination.

The inter-rater reliability analysis conducted produced the Kappa values of 0.742 and 0.815 for title/abstract and full-text screening, respectively. The Kappa values of 0.61 ≤ 0.80 and 0.81 ≤ 1.00 indicated good to very good agreement between independent reviewers, respectively (26). Reviewing the references list of the last 14 full-text articles led to their inclusion in the final analysis. In total, 14 articles met the eligibility criteria for the current scoping review.

### Characteristics of studies

Characteristics of the included studies, such as author, publication year, aims, design, and subjects, are presented in Table 1. The articles reviewed comprised both quantitative and qualitative studies (*n* = 13) focused on health literacy within nursing, with subjects including nurses (*n* = 6), nurse educators (*n* = 1), and nursing students (*n* = 7). Their designs were cross-sectional (*n* = 11), quasi-experimental (*n* = 1), and qualitative (*n* = 1). Each report provided information on health literacy in nursing scopes.

### Synthesis of results

Based on the data synthesis pertaining to the review question shown in Table 2, the synthesis of the results was grouped into two categories. These categories included the role of nurses in health literacy and the factors influencing it.

**Table 2:** Summarized results of the reviewed studies.

**Reference**	**Summarized Results**		
	**Roles of nurses in health literacy**	**Determinant factors**	**Outcomes**
Coşkun and Bebiş (11)	Nurses play a role in facilitating health promotion to increase health literacy and positive health-related lifestyle behaviors	Gender, BMI, family structure, parental knowledge status, income, smoking habit, alcohol drinking habit, history of chronic disease, drug use, and perception of health status	• Health literacy has an impact on increasing the achievement of positive health behaviors. • The ability to find information and media sources related to health increases patient health literacy
Kim and Oh (28)	Nurses facilitate health literacy to carry out health promotion by encouraging the use of social media in seeking information and then increasing information-seeking behavior and self-care agency	Age, gender, education, religion, duration of internet use, and health status	• Electronic health literacy has a significant relationship with health promotion behavior in students. The higher the health literacy, the better the health promotion behavior
Mosley and Taylor (12)	Nurses must have the ability to provide care to patients with low health literacy. Therefore, health literacy education in the nursing process is essential	Age, language, language ability, and risk groups	• Health literacy increases the ability to use social media, online health information-seeking behavior, self-care agency, and health behavior. • Health literacy improves the patient's ability to interpret the disease process, manage their disease, and make decisions regarding health issues.
Shiferaw et al. (29)	The nursing profession must strengthen health literacy skills by increasing access to technology. In the clinical setting, the role of nurses is to improve patients' literacy skills	Gender, domicile, and students' registration years	• Health literacy impacts students' academic abilities or grade point averages. • Meanwhile, the ability to access the internet used to obtain information determines health literacy in college students
Wittenberg et al. (20)	Providing health literacy support to patients is one of the core skills of nursing. Nurses must have good communication skills, specifically in assessing the level of health literacy. This is a strategy to support health literacy	Age, ethnicity, education, income, language, job position, demographic situation, and work experience	Health literacy impacts patient health support and health quality
Ayaz-Alkaya and Terzi (30)	Nurses act as care providers by paying attention to health literacy needs based on knowledge and experience. Every nurse educator must emphasize the importance of health literacy and patient empowerment	Age, gender, family income, social insurance, domicile, history of chronic disease, drug consumption, visual and hearing impairment, and internet use	Health literacy has an impact on increasing patient empowerment. Nurses must have optimal health literacy skills to deal with rapid changes in the health care system
Nesari et al. (19)	Nurses play a role in improving public health literacy practices, such as understanding health information and making decisions about patients' health	Age, work experience, gender, and scope of the work area	• Efforts to increase equity in health literacy are significant in supporting the quality of patient care. • Health literacy practices facilitate effective communication between healthcare professionals and the public
Turan et al. (31)	Nurses can use electronic health literacy and healthy lifestyle behaviors to improve the quality of care by supporting teaching methods and nursing role models. Health literacies are essential to support patients and families in accessing and using health information to improve patient safety and quality of care	Age, health insurance, expenses, health status, and internet usage	• Health literacy is essential in promoting students' healthy lifestyle behavior. • Health literacy positively affects interpersonal relationships, stress management, positive life perspective, spiritual health, and lifestyle.
Koduah et al. (32)	Nursing practice must integrate health literacy knowledge professionally by considering characteristics, social and cultural values, nursing education, and experience	Educational and professional practice factors: nursing specialization, work experience, employment status, length of study, and higher education	Factors of personal characteristics, social and cultural values, nursing education, and experience have a relationship or connection with health literacy knowledge in nursing professional practice
Munangatire et al. (33)	Nurses play an educator role in ensuring patients understand the importance of health literacy and practice to increase their health quality. In the educational setting, the educators ensure that every nurse student understands the health literacy that will impact patients	Age, marital status, gender, and medical history	• Health literacy skills can transfer to students' professional careers as nurses • Nursing students understand health literacy concepts well, but they must all try to translate this understanding into health literacy skills
Yusefi et al. (34)	Nurses play a role in increasing patient health literacy, which affects their quality of life. Health literacy possessed by nurses has an impact on their professional ability to work and decide on a problem	Age, work experience, and employment status	Health literacy's dimensions closely related to the quality of life include access to information, and the ability to read, understand, and use health information
Kim and Oh (35)	Nurses act as health literacy educators for patients by helping to improve their literacy skills that support self-care practices	Age, knowledge, education, communication, patient attitude, and family support	Patient health literacy will increase with the support of effective communication and health education
Yang (36)	Nurses play a role in developing health literacy competencies to improve patient-centered care. Education programs should emphasize the integration of health literacy into the nursing school curriculum	Education level, prior health literacy knowledge, and health literacy competencies	Health literacy skills significantly affect the caring aspect of patients

#### Roles of nurses in health literacy

Nurses play a crucial role in health literacy as caregivers who offer support to enhance patients' health status. As professionals, they need to improve patient health and patient-centered care by providing nursing care that facilitates literacy support. Seven studies stated that nurses have a responsibility of providing health promotion to increase patient health literacy (11, 12, 30, 33–36). Health literacy is an essential factor in the scope of professional nursing practice. According to four studies, it has an impact on health behaviors and promotion that influence patients' health status (11, 28, 31, 32). The impact of health literacy is broad and covers the physical, psychological, social, and spiritual aspects of nursing care practice.

In addition to promoting patient health, providing information and knowledge about the disease is essential. In this case, nurses assist patients in understanding limited information about the disease and treatment procedure. The support of nurses in health literacy has a significant impact on patients' ability to obtain and comprehend disease information and apply it to improve their health behavior (11, 12, 30, 33, 35). With assistance from nurses, patients can interpret the disease process and manage their health effectively (12). The quality of patient care is optimized when nurses provide care that meets their health needs, such as the need for information about the disease being suffered. Three studies found that health literacy affects healthy lifestyle behaviors, as well as improves the quality of life and care (11, 31, 36). Health literacy is a strategy employed by nurses to meet this need.

According to three studies, nurses play an educational role in helping patients understand the value of practicing good health habits and promoting health literacy (30, 33, 35). All nurse educators should emphasize the importance of health literacy skills for themselves and their patients. Another three studies showed that health literacy skills impact the ability of nurses to deal with problems, such as stress management, positive thinking, spiritual wellbeing, and quality of life (11, 31, 34). Therefore, educating patients on health literacy skills becomes crucial to enhance their quality of life and health outcomes. Furthermore, four studies stated that health literacy is one of the core skills of nursing practice (11, 19, 20, 33). Even during the educational process, students must be imparted with health literacy knowledge to be used all through their nursing careers (12, 30, 33).

### Determinant factors of health literacy

Many determinant factors influence health literacy in nursing practice. These include age, gender, education, ethnicity, religiosity, language, income, and marital status, which affect health literacy in both nurses and patients (11–13, 19, 20, 28, 30, 32, 33, 35). Additionally, work experience, work status, information media, and internet usage affect the health literacy of nurses in providing care for patients (19, 20, 28–32, 34). Two studies stated that communication, patient attitudes, and perceptions about their health impact the effectiveness of health literacy in addressing the health problems suffered (11, 35).

Two studies showed the impact of health literacy on health behavior through intermediary mediating factors, such as information-seeking motivation, information-seeking behavior, self-care agency, self-efficacy, and self-care management (13, 28). Another study suggested that health literacy does not directly increase health promotion behavior but directly impacts the use of social media in seeking information, thereby increasing information-seeking behavior and self-care agency (28, 35).

Five studies reported that health literacy in nursing practice is influenced by educational factors and professional training, including nursing specialization or scope of the work area, work experience, employment status, length of study, and educational attainment (19, 29, 32, 34, 37). These factors have an impact on the understanding of health literacy among nurses and their performance in providing care to patients (29, 34, 36).

## Discussion

**Figure 2:**
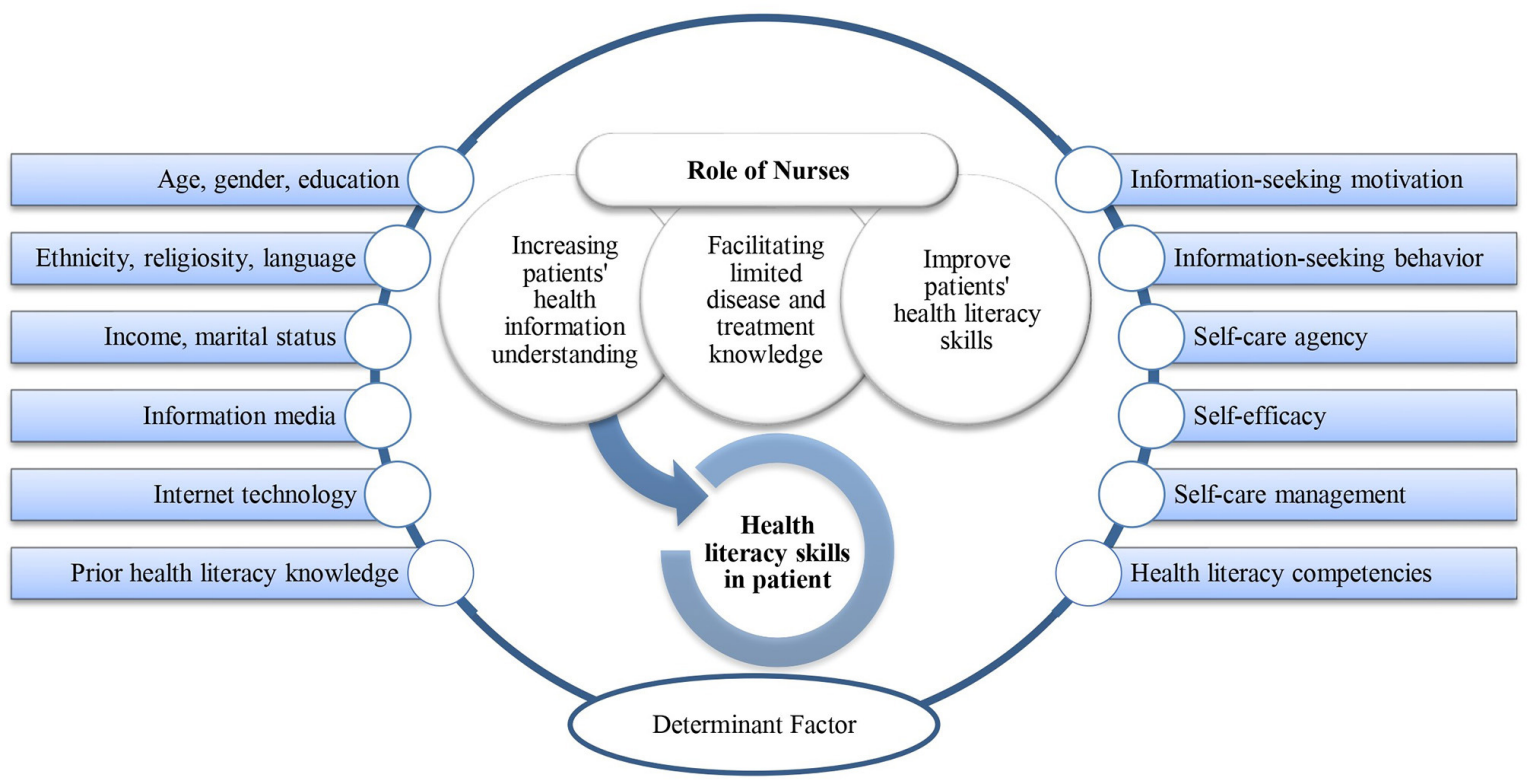
The framework for the role of the nurse and the factors that determine healthy literacy.

This scoping review showed the roles of nurses in patient health literacy as caregivers, facilitators, and educators. As caregivers, they carry out comprehensive care by paying attention to patient health literacy as one of the factors to support the success of treatment goals. To support the success of treatment programs, nurses play a crucial role in enhancing patients' limited knowledge about their disease and treatment options. As educators, nurses provide information, knowledge, and health literacy skills essential for achieving optimal health outcomes. Health literacy is a complex concept and skill in nursing practice influenced by various factors, which nurses and patients should understand to effectively improve the quality of health. This study described the roles of nurses in patient health literacy and the factors influencing the concept within the framework are shown in Figure 2.

A nurse who plays a role in improving health literacy should understand the extent of health literacy skills and patients' level of understanding about a disease or problem. Applying health literacy at any time and in all patient care settings will promote achieving client empowerment, engagement, activation, and optimal health outcomes (38). Nurses have a significant role in educating patients on health information and promotion (12, 35). Their core skill also includes providing support for patient health literacy, which should be understood broadly (37).

Optimizing health outcomes is a fundamental goal of nursing, which involves efforts to prevent and promote health through individual awareness and active engagement in staying. To achieve this, individuals need to possess adequate health literacy skills that ensure the making of good decisions on healthcare, disease prevention, and health promotion, ultimately leading to improved health outcomes (39, 40). Mosley and Taylor described health literacy as an ability required by nurses in adapting care to patients (12). Health literacy in the nursing process requires knowledge, motivation, and competence to access, understand, assess, and apply health information. Nurses play a critical role in facilitating patients' efforts to access and understand health information by developing interventions focused on improving their ability to manage illnesses through comprehensive reading and interpretation of health information (23).

Health literacy in nursing practice has been widely recognized as a mediator between individual and social health status and health outcomes. It is the initial component of obtaining a permanent health culture and optimal health status (41). Health literacy skills are a process to access, understand, evaluate, and use health information, to improve health quality and prevent disease emergence (11). According to the Healthy People 2030 framework, health literacy is the extent to which individuals can find, understand, and use a piece of information to make decisions about their health and that of other individuals (42).

Individuals with good health literacy will be able to participate in public and private discussions about health, medicine, scientific knowledge, and cultural beliefs (43). Ancker's definition places the responsibility of health literacy solely on the individual, leaving the healthcare provider not accountable for providing information in a health-literate manner (44). This situation makes the increase in health literacy not to be optimal. Therefore, strengthening health literacy with the assistance of nurses can enhance primary health conditions at the individual and community levels by using appropriate health information to increase health literacy and meet patient needs (12, 20).

Health literacy is how individuals can use the information studied effectively to determine the most appropriate nursing intervention offered to patients. Nurses are expected to assess the level of health literacy of patients (20). Decisions made based on health literacy are highlighted discoveries because being “well-informed” is not just the right decision. Therefore, health literacy is a process of obtaining and using appropriate information in deciding about personal health and the health of others. Nurses can utilize health literacy strategies in informing individuals to make the best possible health-related decision.

In nursing, health literacy is essential in communication strategies for all clients (35). Clients cannot fully participate in their healthcare choices or make appropriate decisions without accurate information for their health literacy needs (45). Nurses as healthcare workers who have a closer relationship with patients can become facilitators to meet these health literacy needs through health education or promotion activities. Sometimes nurses overestimate patients' level of health literacy, by creating reports based on their feelings and relying only on external indicators such as education or socioeconomic status. This approach is problematic because health literacy cannot be determined solely with the aforementioned factors. While providing information to the patients, nurses should be careful in interpreting their observable body language movements, such as nodding of the head as an indication that the information received was understood (46).

As previously explained, health literacy is a complex concept that requires careful, thorough, and persistent assessment. Inadequate assessment of health literacy can pose a significant barrier to clients' health literacy. As stated by Cohen et al. (47), to ensure understanding and overcome health literacy barriers, nurses must conduct adequate assessments to determine patients' level of knowledge, emotional reactions to information, and external support from other healthcare teams.

Wittenberg et al. (37) provided several recommendations for assessing health literacy in individuals, groups, or communities experiencing barriers. This includes the following: (1) Nurses must understand the beliefs and cultural norms of their clients. (2) Information explanations should be repeated or provided in a way that is more technically adapted to clients' cultural approach. (3) Nurses should be attentive to verbal and non-verbal cues, and use simple language during communication, avoiding medical terms that may be unfamiliar to clients. (4) In case of a language barrier, a translator should be involved to ensure effective communication.

Assessing patient understanding and addressing challenges related to health literacy are essential for providing quality nursing care and improving health literacy. Therefore, nurses should possess good health literacy knowledge and experience to empower patients with the best information for their health. They need to emphasize the importance of health literacy and patient empowerment to achieve effective care.

Various determinant factors influence health literacy skills, which is the basis for professional nursing practice in supporting patient care. These should be considered by interventions meant to improve health status through good health literacy in individuals, communities, and society. Personal factors, specifically age, gender, income, education, and employment status, are associated with the determination of health literacy (11, 13, 20, 28, 30, 32, 33, 35). Among all, age has the closest relationship with health literacy as they are both inversely proportional (48–50). Similarly, Sántha (14) discovered that health literacy has a significant correlation with age and education.

According to Yang et al. (50), personal factors and access to information or its sources, such as the internet, play a crucial role in determining an individual's health literacy. Health literacy is a concept based on information existence, hence inadequate access to information may hinder health literacy development, despite possessing literacy skills. Various studies found that using multiple sources of information significantly impacts an individual's level of health literacy (28, 30, 31). Motivational factors and the ability to access information can influence health literacy (51).

In contrast to some previous studies, Guo et al. (39) found that sociodemographic factors, including gender, education, income, and access to information, are not directly related to health literacy. Personal characteristics, such as self-efficacy, social support, and health status, were observed to have a significant impact on health literacy. Furthermore, health status has been identified as a key predictor of health literacy. This along with factors such as disease history, disease severity, presence of other disorders, and the use of drugs can affect patients' ability to improve or decrease their health literacy (11, 13, 28, 30, 31, 33).

Health literacy is also affected by spiritual and environmental factors, as well as an individual's ecological aspects, education, place of work, and religious beliefs (8). The study conducted by Koduah et al. (32) discovered that religiosity can impact health literacy. Nurses with strong religious beliefs view health as necessary and make health literacy essential to their lives. Home and work environments can impact health literacy (19, 20, 32, 34). Therefore, nurses play a crucial role in improving and determining the health literacy levels of individuals, groups, or communities by considering these factors (20, 30, 32).

### Strengths and limitations

This review systematically outlines the peer-reviewed literature related to health literacy within the scope of nursing science. Although health literacy skills are essential for nurses at all levels, specifically those providing direct care to patients, this study does not explicitly focus on any particular level. The literature analyzed was various study types, and the evidence level obtained was not the highest. Books, gray literature, or other sources that can provide more in-depth information on the subject of health literacy were not included in this scoping review.

## Conclusion

The results showed that nurses play an essential role in supporting health literacy through the dissemination of relevant information. Health literacy is one of the critical skills that nurses and patients must master to make informed decisions capable of impacting health outcomes. Nurses act as caregivers, facilitators, and educators in meeting the limited health literacy needs of patients to improve their health status. They help to optimize patients' health literacy skills by facilitating the ability to find, understand, assess, and use health information related to their diseases. It is important to note that health literacy is influenced by various factors, and nurses should utilize these factors as opportunities to promote health literacy improvement. By fully understanding health literacy from a nursing perspective, nurses can use this approach to solve health problems and ultimately improve the quality of care for patients.

## Author contributions

AW and MP created the presented idea and then revised the manuscript written. AW wrote the draft of the manuscript, directed the study, served as the guarantor responsible for the study's conduct and finished work, and had access to the data collected. MP and AY performed the review. All authors discussed the results and provided feedback on the manuscript.
